# Internet Addiction and Relationships with Insomnia, Anxiety, Depression, Stress and Self-Esteem in University Students: A Cross-Sectional Designed Study

**DOI:** 10.1371/journal.pone.0161126

**Published:** 2016-09-12

**Authors:** Farah Younes, Ghinwa Halawi, Hicham Jabbour, Nada El Osta, Latife Karam, Aline Hajj, Lydia Rabbaa Khabbaz

**Affiliations:** 1 Faculty of Pharmacy, Saint-Joseph University of Beirut, Beirut, Lebanon; 2 Department of Anesthesia and Critical Care, Hôtel-Dieu de France Hospital, Saint-Joseph University of Beirut, Beirut, Lebanon; 3 Faculty of Medicine, Saint-Joseph University of Beirut, Beirut, Lebanon; 4 Department of Public Health, Faculty of Medicine, Saint-Joseph University, Beirut, Lebanon; 5 Department of Prosthodontics, Faculty of Dental Medicine, Saint-Joseph University, Beirut, Lebanon; 6 Clermont University, University of Auvergne, CROC-EA4847, Centre de Recherche en Odontologie Clinique, BP 10448, F-63000, Clermont-Ferrand, France; 7 Pharmacy department, Hôtel-Dieu de France Hospital, Saint-Joseph University of Beirut, Beirut, Lebanon; 8 Laboratoire de Pharmacologie, Pharmacie clinique et Contrôle de qualité des médicaments, Faculty of Pharmacy, Saint-Joseph University of Beirut, Beirut, Lebanon; University of Rome Tor Vergata, ITALY

## Abstract

**Background and Aims:**

Internet addiction (IA) could be a major concern in university medical students aiming to develop into health professionals. The implications of this addiction as well as its association with sleep, mood disorders and self-esteem can hinder their studies, impact their long-term career goals and have wide and detrimental consequences for society as a whole. The objectives of this study were to: 1) Assess potential IA in university medical students, as well as factors associated with it; 2) Assess the relationships between potential IA, insomnia, depression, anxiety, stress and self-esteem.

**Methods:**

Our study was a cross-sectional questionnaire-based survey conducted among 600 students of three faculties: medicine, dentistry and pharmacy at Saint-Joseph University. Four validated and reliable questionnaires were used: the Young Internet Addiction Test, the Insomnia Severity Index, the Depression Anxiety Stress Scales (DASS 21), and the Rosenberg Self Esteem Scale (RSES).

**Results:**

The average YIAT score was 30 ± 18.474; Potential IA prevalence rate was 16.8% (95% confidence interval: 13.81–19.79%) and it was significantly different between males and females (*p*-value = 0.003), with a higher prevalence in males (23.6% versus 13.9%). Significant correlations were found between potential IA and insomnia, stress, anxiety, depression and self-esteem (*p*-value < 0.001); ISI and DASS sub-scores were higher and self-esteem lower in students with potential IA.

**Conclusions:**

Identifying students with potential IA is important because this addiction often coexists with other psychological problems. Therefore, interventions should include not only IA management but also associated psychosocial stressors such as insomnia, anxiety, depression, stress, and self-esteem.

## Introduction

Internet use has grown exponentially worldwide to more than 2.5 billion active users [1, 2] with the majority being adolescents and young people [3]. Paralleling the rapid growth in internet access is a rise in internet addiction, especially among adolescents, gaining increased attention from the popular media, government authorities, and researchers [4].

Excessive internet use is defined as when internet use has become excessive, uncontrolled, and time-consuming to the point of timelessness and severely disrupting people’s lives [5]. Internet addiction is characterized by a maladaptive pattern of internet use leading to clinically significant impairment or distress [6].

The terms “problematic internet use” [7], pathological internet use [8–10] and “internet addiction” [11–13] are usually considered synonyms of internet dependence [14]. Young et al [15–17] proposed diagnostic criteria for internet addiction (IA) in which withdrawal, poor planning abilities, tolerance, preoccupation, impairment of control, and excessive online time were defined as core symptoms.

Worldwide prevalence of IA ranged from 1.6%-18% [18]. 10.7% of adolescents in South Korea present IA according to Yong’s internet addiction scale [19]. 11% in Greece, based on the same test [20]; 10.7–13.9% of European adolescents are at-risk for addictive use, based on Young's instruments [21] and 4% in high school students in the USA [22].

IA prevalence may vary according to age, sex and ethnicity, and it prevails more commonly among college students [23].

A high rate of personality disorders are found in individuals with IA [24–27].

Heavy internet use was also reported to be associated to mood disorders [28], poor sleep quality [28, 29], low self-esteem [30], impulsivity [31], suicide [32, 33], lower levels of physical activity [29], and health problems (migraines, back pain, obesity) [34].

Our hypothesis were that IA could be a major concern in university medical students, and that examining its association with sleep, mood disorders and self-esteem is important, so that appropriate measures can be taken to address this issue.

For medical students aiming to develop into health professionals, the implications of this addiction can hinder their studies and impact their long-term career goals and can have wide and detrimental consequences on society as a whole.

The objectives of this study were to: 1) Assess potential IA in students at the Campus of Medical Sciences (CMS) at Saint-Joseph University in Lebanon, as well as socio-demographic factors associated with it; 2) Assess the relationships between potential IA, insomnia, depression, anxiety, stress and self-esteem while accounting for the simultaneous exposure to insomnia, stress, anxiety and depression in students.

## Materials and Methods

### Ethical considerations

The protocol of the study was approved by the ethics committee of Saint-Joseph University (Ref USJ-2015-28, June 2015). Informed written consent was obtained from all individuals participating in the study.

### Survey procedure and sampling

Our study was a cross-sectional questionnaire-based survey conducted among students of three faculties: medicine, dentistry and pharmacy at Saint-Joseph University, from September to December 2015 (4 months). Inclusion criteria were: students aged 18 years and above, and willing to participate in the study. Exclusion criteria were: age under 18 years and presence of a chronic disease. Students were randomly selected within each class using a random number table to ensure the representativeness of the sample. This random selection was proportional to the number of students in each class. Students selected were approached by two trained research assistants usually at the end of their courses before leaving the classroom and asked if they were willing to participate on the condition that they didn’t present any exclusion criteria. A written formal consent was then obtained.

### Data collection

Data were collected during a face-to-face interview using a self-administered standardized survey tool based on four internationally validated and reliable questionnaires, namely the Young Internet Addiction Test, the Insomnia Severity Index, the Depression Anxiety Stress Scales (DASS 21), and the Rosenberg Self Esteem Scale. The duration of interviews ranged from 15 to 25 minutes.

### Measures

#### Participants

Personal data about age, gender and faculty were collected. Furthermore, information about living alone or not, tobacco (cigarette or water pipe), and alcohol use were also obtained.

#### Internet addiction

The Young Internet Addiction Test (YIAT) is validated among adolescents and adults and widely used [15, 16, 35]. It is a 20-item self-report scale assessing a respondent’s productivity at work, school, or home (3 questions), social behaviors (3 questions), emotional connection to and response from using the internet (7 questions), and general patterns of Internet use (7 questions). Participants respond to the 20 YIAT items on a 6-point Likert measure (“does not apply” to “always”), which produced an overall score between 0 and 100. The following cut-off points to the total YIAT score were applied: (1) normal internet use: scores 0–49 and (2) potential internet addiction: scores over 50 [36, 37].

#### Insomnia

The ISI is a 7-item self-report questionnaire assessing the nature, severity, and impact of insomnia. The evaluated domains are: severity of sleep onset, sleep maintenance, early morning awakening problems, sleep dissatisfaction, interference of sleep difficulties with daytime functioning, perception of sleep difficulties by others, and distress caused by the sleep difficulties. A 5-point Likert scale was used to rate each item (0 to 4 where 0 indicates no problem and 4 corresponds to a very severe problem), yielding a total score ranging from 0 to 28. The total score was interpreted as follows: absence of insomnia (0–7); sub-clinical or mild insomnia (8–14); moderate insomnia (15–21); and severe insomnia (22–28). Furthermore, clinically significant insomnia was detected when the total score was >14 [38, 39].

#### Self-esteem

The Rosenberg Self Esteem Scale (RSES) is commonly used and its internal consistency and reliability were confirmed in many previous studies [40]. It comprises 10 statements. Participants rate the extent to which they agree with each statement on a four-point Likert scale, (0) strongly disagree to (3) strongly agree for items 1, 2, 4, 6 and 7 and opposite rating for items 3, 5, 8, 9 and 10. A total score is obtained by summing all responses and may range from 0 to 30, with higher scores indicating higher self-esteem [41].

#### Anxiety, depression and stress

The Depression Anxiety Stress Scales (DASS) is a widely used measure of negative affect in adults [42]. An important and unique feature of the DASS is its inclusion of a tension/Stress scale in addition to the depression and anxiety scales. The DASS 21 is a short version of the 42-item original scale. Both are reliable and valid measures of depression, anxiety and tension/stress in clinical and non-clinical populations of adults [43–45].

It is a 21-item scale measured on a 4-point Likert scale (0–3), “0” denoting “did not apply to me at all” and “3” denoting “applied to me very much, or most of the time”.

The following cut-off scores are used for each subscale: depression: normal 0–4, mild 5–6, moderate 7–10, severe 11–13 and extremely severe 14+; anxiety: normal 0–3, mild 4–5, moderate 7–10, severe 11–13 and extremely severe 10+; stress: normal 0–7, mild 8–9, moderate 10–12, severe 13–16 and extremely severe 17+.

#### Statistical analysis

The statistical analysis was carried out using SPSS software for Windows (version 18.0, Chicago, IL, USA). The significance level was set at 0.05. Sample characteristics were summarized using the mean and the standard deviation (SD) for continuous variables and percentage for categorical variables. Insomnia and internet addiction prevalence rates were calculated using descriptive data, along with corresponding 95% confidence interval (CI). The Kolmogorov-Smirnov tests were used to assess the normality of the distribution of each variable.

Internet addiction categories were grouped as normal internet users and potential internet addiction.

Multivariate analysis were required to determine the impact of multiple explicative explanatory variables presented simultaneously and to determine which of the explanatory factors act independently on the internet addiction.

In the initial stages, the univariate analysis of categorical and continuous variables were carried out using respectively the Chi-square independence tests or Fisher Exact test and the Student’s t-test or Mann-Whitney test. Subsequently, logistic regression analysis were performed with the dichotomized internet addiction (<50, ≥50) as the dependent variable. Participants’ characteristics and scores (ISI, DASS A, DASS S, DASS D, RSES) that showed associations with p-value <0.25 in univariate analysis, were candidates for the multivariate model, according to the Enter method. Collinearity among independent variables was also tested. Independent variables highly correlated were excluded.

It has been suggested not to include two independent variables where there is a correlation of 0.64 or more. Anxiety, stress and depression were not entered in the same model, since they were highly correlated together, indicated by the Spearman and Pearson correlation coefficients. Finally, three logistic regression analysis were conducted and the independent variables included in the model were gender, tobacco smoking, ISI score, RSES score and the DAS score for stress, anxiety and depression in each of the three model.

## Results

### Socio-demographic characteristics of the participants

A total of 780 students were approached to participate in the study, of whom 600 (77%) consented. Our study population comprised 182 (30.3%) male and 418 (69.7%) female students. Age ranged between 18 and 28 years with a mean of 20.36 ± 1.83 years.

The sample included 219 students from the Faculty of medicine (FM), 109 from the Faculty of dentistry (FD) and 272 from the Faculty of pharmacy (FP). Table 1 summarizes participants’ characteristics.

**Table 1:** Participants’ characteristics (N = 600).

**Gender**	Male	182(30.30%)
	Female	418(69.70%)
**Age (Mean ± SD)**		20.36 ± 1.84
**Living alone**	Yes	91(15.20%)
	No	509(84.80%)
**Cigarette smoking**	Yes	25(4.20%)
	No	574(95.70%)
	Missing value	1(0.20%)
**Number of cigarettes per day (n = 21)**		9.21 ±11.78
**Smoking since (years) (n = 23)**		3.02 ±1.85
**Water pipe**	Yes	52(8.70%)
	No	547(91.20%)
	Missing value	1(0.20%)
**Number of water pipe smoking per week (n = 45)**		1.90 ±1.91
**Smoking since (years) (n = 39)**		3.03 ±1.97
**Alcohol**	Yes	209(34.80%)
	No	390(64.00%)
	Missing value	1(0.20%)
**Alcohol intake frequency**	Occasionally	45(7.5%)
	Once / week	121(20.17%)
	Twice/week	31(5.17%)
	3 times / week	6(1.00%)
	4 times / week	1(0.17%)
	Missing values	6(1.00%)

### Internet addiction prevalence (YIAT)

The average YIAT score was 30 ± 18.47 (Table 2); Potential internet addiction prevalence rate was 16.80% with a 95% CI of 13.81–19.79%. “S1 Table” summarizes average scores for each of the 20 items of the YIAT.

**Table 2:** Number and percentage of students in each category of the three questionnaires: ISI, DASS and YIAT with mean (SD) scores (N = 600).

**ISI**		**N(%)**
	No insomnia	209(34.80%)
	Mild insomnia	332(55.30%)
	Moderate insomnia (clinically significant)	59(9.80%)
	**Mean score (SD)**	**9.31 (3.76)**
**DASS S**		**N(%)**
	Normal	199(33.20%)
	Mild	107(17.80%)
	Moderate	162(27.00%)
	Severe	75(12.50%)
	Extremely severe	57(9.50%)
	**Mean score (SD)**	**6.99(4.46)**
**DASS A**		**N(%)**
	Normal	268(44.70%)
	Mild	107(17.80%)
	Moderate	94(15.70%)
	Severe	62(10.30%)
	Extremely severe	69(11.50%)
	**Mean score (SD)**	**4.77(3.79)**
**DASS D**		**N(%)**
	Normal	434(72.30%)
	Mild	54(9.00%)
	Moderate	64(10.70%)
	Severe	33(5.50%)
	Extremely severe	15(2.50%)
	**Mean score (SD)**	**5.43(4.43)**
**YIAT**		**N(%)**
	Normal internet use	499(83.20%)
	Potential internet addiction	101(16.80%)
	**Mean score (SD)**	**30(18.47)**

#### Univariate analysis

The univariate analysis showed that potential internet addiction was significantly different between males and females (p-value = 0.003), with a higher prevalence in males (23.60% versus 13.90%). Tobacco smoking was significantly related to potential internet addiction (p-value = 0.046); however, neither age, faculty, regular alcohol intake, nor living alone, was significantly related to internet use (Table 3).

**Table 3:** Univariate analysis of the relationships between potential internet addiction and participants’ characteristics (N = 600).

		Normal Internet use	Potential Internet addiction	*p*-value
**Gender**				0.003*
	Male	139(76.40%)	43(23.60%)	
	Female	360(86.10%)	58(13.90%)	
**Age (mean ± SD)**		20.36 ± 1.83	20.35 ± 1.84	0.932**
**Faculty**				0.576*
	Pharmacy	229(84.20%)	43(15.80%)	
	Medicine	183(83.60%)	36(16.40%)	
	Dentistry	87(79.80%)	22(20.20%)	
**Year of study**				0.352***
	First	83(85.60%)	14(14.40%)	
	Second	116(77.90%)	33(22.10%)	
	Third	77(84.60%)	14(15.40%)	
	Fourth	81(89.00%)	10(11.00%)	
	Fifth	108(83.70%)	21(16.30%)	
	Sixth	18(81.80%)	4(18.20%)	
	Seventh	16(76.20%)	5(23.80%)	
**Living alone**				0.836*
	Yes	424(83.30%)	85(16.70%)	
	No	75(82.40%)	16(17.60%)	
**Tobacco smoking**				**0.046**^$^
	Yes	51(75.00%)	17(25.00%)	
	No	447(84.20%)	84(15.80%)	
**Alcohol regular intake**				0.873***
	Yes	**137(83.50%)**	**27(16.50%)**	
	No	**361(83.00%)**	**74(17.00%)**	

*Chi two test bilateral

**Mann-Whitney

***Fisher exact test

^$^ Chi Square test

Numbers in bold are for significant *p*-values

### Insomnia prevalence and severity (ISI)

Insomnia was evaluated according to the ISI questionnaire. The mean ISI score of the sample was 9.31 ± 3.76. Prevalence of clinically significant insomnia was 9.80% with a 95% CI ranging between 7.42 and 12.18% (Table 2).

### Anxiety, depression and stress (DASS-21)

Anxiety: DASS A. The average DASS A score was 4.77 ± 3.79. 44.70% of participants presented a normal DASS A score (Table 2).

Depression: DASS D. The average DASS D score was 5.43 ± 4.43. The majority of the participants presented a normal DASS D score (Table 2).

Stress: DASS S. The average DASS S score was 6.99 ± 4.46 and 33.20% of participants presented a normal DASS S score (Table 2).

### Self-esteem (RSES)

The average RSES score of the study sample was 22.63 ± 5.29 (S file).

### Associations between internet addiction, insomnia, low self-esteem, anxiety and depression

A significant relationship was found between potential internet addiction and insomnia (*p*-value < 0.00001) (Table 4).

**Table 4:** Univariate analysis of the relationships between the questionnaires’ scores (N = 600).

		ISI	DASS S	DASS A	DASS D	YIAT
**ISI**	Correlation Coefficient	1.000	.404	.367	.366	.268
	*p*-value	**.**	**.000**	**.000**	**.000**	**.000**
	N	600	600	600	600	600
**DASS S**	Correlation Coefficient	.404	1.000	.669	.715	.316
	*p*-value	**.000**	**.**	**.000**	**.000**	**.000**
	N	600	600	600	600	600
**DASS A**	Correlation Coefficient	.367	.669	1.000	.643	.350
	*p*-value	**.000**	**.000**	**.**	**.000**	**.000**
	N	600	600	600	600	600
**DASS D**	Correlation Coefficient	.366	.715	.643	1.000	.327
	*p*-value	**.000**	**.000**	**.000**	**.**	**.000**
	N	600	600	600	600	600
**YIAT**	Correlation Coefficient	.268	.316	.350	.327	1.000
	*p*-value	**.000**	**.000**	**.000**	**.000**	**.**
	N	600	600	600	600	600
**RSES**	Correlation Coefficient	-.238	-.334	-.371	-.512	-.286
	*p*-value	**.000**	**.000**	**.000**	**.000**	**.000**
	N	600	600	600	600	600

Numbers in bold represent significant results.

The average ISI score was 8.99 ± 3.65 for normal internet users versus 10.89 ± 3.90 in the potential internet addiction group (p<0.0001) (Table 5).

**Table 5:** Univariate analysis of the relationships between ISI, DASS A, DASS S, DASS D, and RSES scores and potential internet addiction (N = 600).

		N	Mean ± sd	*p*-value
**ISI total**				**< 0.001**
	Normal internet users	499	8.99 ± 3.65	
	Potential internet addiction	101	10.89 ± 3.90	
**DASS S**				**< 0.001**
	Normal internet users	499	6.50 ± 4.30	
	Potential internet addiction	101	9.41 ± 4.45	
**DASS A**				**< 0.001**
	Normal internet users	499	4.30 ± 3.53	
	Potential internet addiction	101	7.09 ± 4.17	
**DASS D**				**< 0.001**
	Normal internet users	499	4.91 ± 4.19	
	Potential internet addiction	101	8.02 ± 4.68	
**RSES**				**< 0.001**
	Normal internet users	499	23.05 ± 5.152	
	Potential internet addiction	101	20.15 ± 5.33	

Mann-Whitney test; numbers in bold represent significant results.

Moreover, a significant relationship was found between potential internet addiction and anxiety, depression and stress (Tables 4 and 5). DASS average scores were significantly higher in the potential internet addiction group for anxiety, depression and stress.

As for self-esteem, a significant correlation was found between YIAT and RSES scores with low self-esteem being associated to potential internet addiction (Tables 4 and 5).

### Logistic regression model

The logistic regression model showed that gender, ISI, DASS A, S and D, and RSES scores were significantly associated with internet addiction. Once the explicative variables were controlled for in multivariate analysis, the association between tobacco smoking and internet addiction was no longer significant (p > 0.05), (Table 6).

**Table 6:** Multivariate analysis of the relationships between internet addiction and gender, tobacco smoking, ISI, RSES, DASS A, DASS S, and DASS D scores (N = 600).

Independent variables	B	S.E.	df	Sig.	OR	95.0% CI for OR	
						Lower	Upper
Gender	.872	.246	1	.000	2.393	1.476	3.878
Tobacco smoking	-.359	.331	1	.278	.699	.365	1.336
ISI	.082	.033	1	.013	1.085	1.018	1.158
RSES	-.052	.023	1	.021	.949	.908	.992
DASS A	.146	.032	1	.000	1.157	1.087	1.232
Constant	-2.598	1.015	1	.010	.074		
Independent variables	B	S.E.	df	Sig.	OR	95.0% CI for OR	
						Lower	Upper
Gender	.760	.241	1	.002	2.139	1.334	3.429
Tobacco smoking	-.417	.325	1	.199	.659	.348	1.246
ISI	.082	.033	1	.013	1.085	1.018	1.157
RSES	-.061	.022	1	.005	.940	.901	.982
DASS S	.106	.028	1	.000	1.112	1.052	1.174
Constant	-2.163	.988	1	.029	.115		
Independent variables	B	S.E.	df	Sig.	Exp(B)	95.0% CI for OR	
						Lower	Upper
Gender	.792	.241	1	.001	2.208	1.375	3.544
Tobacco smoking	-.360	.327	1	.270	.698	.368	1.323
ISI	.087	.032	1	.007	1.091	1.024	1.163
RSES	-.043	.024	1	.071	.958	.914	1.004
DASS D	.107	.029	1	.000	1.113	1.052	1.178
Constant	-2.609	1.034	1	.012	.074		

## Discussion

We aimed to determine the prevalence of potential IA in Lebanese university medical students, to evaluate relationships between IA and participants’ characteristics (mainly age, gender, smoking habits, alcohol intake), and to explore possible associations between IA, insomnia, anxiety, depression, stress, and self-esteem.

Our study revealed that potential IA was significantly related to gender and higher among males. 16.80% of participants suffered from potential IA, with a mean YIAT score of 30. These results are comparable to those previously reported for young adults [1, 4, 6, 13]. Some studies reported that IA prevalence was higher in males [46], while others did not find difference between genders [34].

When examining insomnia, our results also showed that 9.8% of participants suffered from clinically significant insomnia and a strong correlation was found between potential internet addiction and insomnia. Insomnia prevalence reported in this study is consistent with the nature of the sample studied (young students) and is comparable to what is reported in young adults aged 20 to 29 (9.1%) [47, 48] and in college students (12–13%) [49].

Sleep problems are usually considered negative outcomes or complications of internet addiction [50], but reverse causation is also possible since sleep problems predicted a longer time spent on social networking sites among young university students [51]. In a systematic review of the literature, addictive gaming was found to be associated to poorer sleep quality and problematic internet use was associated with subjective insomnia and poor sleep quality [52]. However, study designs as well as questionnaires used were very heterogeneous and it was mainly sleep quality that was explored, much less insomnia.

Furthermore, a strong correlation was found in this study between potential internet addiction and anxiety, stress, and depression: the percentage of students suffering from anxiety, depression or stress is higher among potential internet addicts. Previous published studies have already indicated a potential correlation between pathological internet use and depression [53, 54] and anxiety [55]; however, data has been contradictory [56] and studies examined pathological internet use and not addiction as defined by Young.

Finally, an important finding of our study was that self-esteem is significantly related to internet addiction as well as to the psychological profile of students: RSES scores were inversely correlated to ISI, DASS A, DASS S, DASS D and YIAT scores. A decrease in self-esteem seems associated to increased insomnia, anxiety, depression, stress and to potential IA.

Self-esteem is described as the evaluation one has of his/herself, how one feels about his/herself in almost all situations [40, 41]. When social integration and support are low, the level of self-esteem will accordingly decrease [57].

Detecting factors associated to low esteem in students is of considerable importance because an inverse relation exists between self-esteem and depression and anxiety [58, 59] and the decrease in the feeling of self-esteem may lead to an increase in suicidal ideation [60].

### Strength and limitations

Our findings should be interpreted in the context of the study’s design and limitations. The results of our survey rely on self-reported behavior. Self-reporting questionnaires remain the most widely used tools in community surveys for physical and mental health evaluation [61, 62, 63]. The self-report method reflects the interviewee’s own perspective, which may be more suitable for reporting subjective disorders. The questionnaires were formulated in a “multiple-choice” and scale pattern to facilitate response and have shorter interview duration in order to avoid disturbing the students, in the hope that the simplicity of the questionnaire would make it easier for the respondents to give accurate information. Chronic use of drugs was not assessed since the presence of any chronic disease was among the exclusion criteria of this study. Finally, the study did not examine the repercussion of internet addiction on achievements, in terms of grades, failure or success, which could have been interesting.

Notwithstanding these limitations, the findings observed in this study are important and warrant further investigations.

To the best of our knowledge, this was the first study that assesses the relation between five different psychosocial stressors: insomnia, anxiety, depression, stress, self-esteem, and IA in university students.

Our findings denote the importance of identifying and offering help to students with potential IA because this addiction often coexists with other psychological problems, and IA could be one visible tip of a complex iceberg.

## Supporting Information

S1 Table This is the individual and complete data for all participants (SPSS sheet). (DOCX)
