# Supplementary material for: Internet Addiction and Relationships with Insomnia, Anxiety, Depression, Stress and Self-Esteem in University Students: A Cross-Sectional Designed Study
Source: PLoS One. 2016 Sep 12;11(9):e0161126. doi: 10.1371/journal.pone.0161126 (PMC5019372; doi:10.1371/journal.pone.0161126)
Supplement: S1 Table — (DOCX) [file pone.0161126.s001.docx]

**S1 Table. Range, mean score ± sd for each of the 20 items of the YIAT (N=600).**

|  | **Normal Internet use**  **n=499** | | **Potential Internet Addiction**  **n=101** | |
| --- | --- | --- | --- | --- |
| **Items** | **Range** | **Mean ± sd** | **Range** | **Mean ± sd** |
| 1 | 0-5 | 2.71 ± 1.48 | 1-5 | 4.23 ± .90 |
| 2 | 0-5 | 1.86 ± 1.38 | 0-5 | 3.80 ± 1.18 |
| 3 | 0-5 | 0.64 ± .97 | 0-5 | 2.03 ± 1.63 |
| 4 | 0-5 | 0.73 ± 1.04 | 0-5 | 2.31 ± 1.77 |
| 5 | 0-5 | 1.38 ± 1.31 | 0-5 | 3.22 ± 1.43 |
| 6 | 0-5 | 1.00 ± 1.17 | 0-5 | 3.03 ± 1.46 |
| 7 | 0-5 | 1.85 ± 1.47 | 0-5 | 3.59 ± 1.27 |
| 8 | 0-5 | 1.03 ± 1.07 | 0-5 | 2.92 ± 1.35 |
| 9 | 0-5 | 1.18 ± 1.20 | 0-5 | 3.06 ± 1.31 |
| 10 | 0-5 | 1.21 ± 1.27 | 0-5 | 3.06 ± 1.42 |
| 11 | 0-5 | 1.22 ± 1.19 | 0-5 | 3.15 ± 1.39 |
| 12 | 0-5 | 1.43 ± 1.37 | 0-5 | 3.11 ± 1.22 |
| 13 | 0-5 | 0.99 ± 1.09 | 0-5 | 2.99 ± 1.31 |
| 14 | 0-5 | 1.50 ± 1.36 | 0-5 | 3.62 ± 1.29 |
| 15 | 0-5 | 0.52 ± .85 | 0-5 | 2.38 ± 1.53 |
| 16 | 0-5 | 1.90 ± 1.47 | 0-5 | 3.81 ± 1.25 |
| 17 | 0-5 | 1.11 ± 1.20 | 0-5 | 3.11 ± 1.52 |
| 18 | 0-5 | .54 ± .92 | 0-5 | 2.37 ± 1.55 |
| 19 | 0-4 | .49 ± .81 | 0-5 | 2.18 ± 1.51 |
| 20 | 0-5 | .58 ± .88 | 0-5 | 2.24 ± 1.47 |
